# Robotic quantification of the mechanical effect of corrective manoeuvres for intraoperative extension deficiency in total knee arthroplasty: A cadaveric study

**DOI:** 10.1002/jeo2.70740

**Published:** 2026-05-08

**Authors:** Albert Pons‐Riverola, Juan Ignacio Erquicia, Eric Camprubí, Berta Gasol, Ángela Zumel, Santiago Bonduel, Joan Leal‐Blanquet

**Affiliations:** ^1^ Department of Orthopedic Surgery and Traumatology Althaia Xarxa Assistencial Universitària de Manresa Manresa Spain; ^2^ MOVE Traumatologia, Mi Tres Torres Barcelona Spain; ^3^ Department of Orthopedic Surgery and Traumatology Hospital Fernández Buenos Aires Argentina

**Keywords:** bone resection, fixed flexion contracture, robotic knee arthroplasty, soft tissue release, total knee arthroplasty

## Abstract

**Purpose:**

Intraoperative extension deficiency (IED) may arise after trial component implantation during total knee arthroplasty (TKA), a condition conceptually distinct from preoperative fixed flexion contracture (FFC). This study utilised a robotic system to quantify the incremental mechanical effect of sequential surgical actions—posterior capsule release (PCR), posterior cruciate ligament (PCL) excision and incremental distal femoral resections—to correct IED. A secondary objective was to evaluate their impact on manually assessed mediolateral laxity.

**Methods:**

Twenty‐nine robotic TKAs were performed on 15 fresh‐frozen cadaveric specimens. Nineteen knees presented with IED. All knees underwent PCR as the initial corrective manoeuvre. Subsequently, a quasi‐experimental design based on laterality was employed to evaluate two sequences: right knees underwent PCL excision followed by up to four 1‐mm distal femoral recuts; left knees followed the reverse order. Extension gains and manually assessed mediolateral laxities were recorded after each step.

**Results:**

Baseline IED averaged 6.6° ± 4.9°. Posterior capsule release corrected IED in 36.8% of the knees, yielding a mean extension gain of 1.68° ± 1.11°. The mechanical impact of distal femoral resections varied depending on PCL status: when the PCL remained intact, distal femoral recuts produced a substantial mean gain of 4.3° ± 0.6° per millimetre; however, following PCL excision, the gain per millimetre decreased to 2.8° ± 0.8°. PCL excision alone improved extension by a mean of 1.1° ± 1.3°. No significant changes in manually assessed mediolateral laxity at 0° and 90° of flexion were observed across manoeuvres.

**Conclusions:**

Robotic quantification demonstrates that the mechanical effectiveness of distal femoral resection is significantly enhanced by an intact PCL. While PCR is an impactful initial step for mild IED, distal femoral recuts provide greater extension gains. These objective data provide the foundation for a structured strategy to guide the intraoperative management of IED in both robotic and conventional TKA.

**Level of Evidence:**

N/A (cadaveric).

## Graphical abstract

Precise robotic quantification of extension gains and joint laxity impacts from sequential maneuvers yields a data‐driven, structured strategy to manage intraoperative extension deficiency.

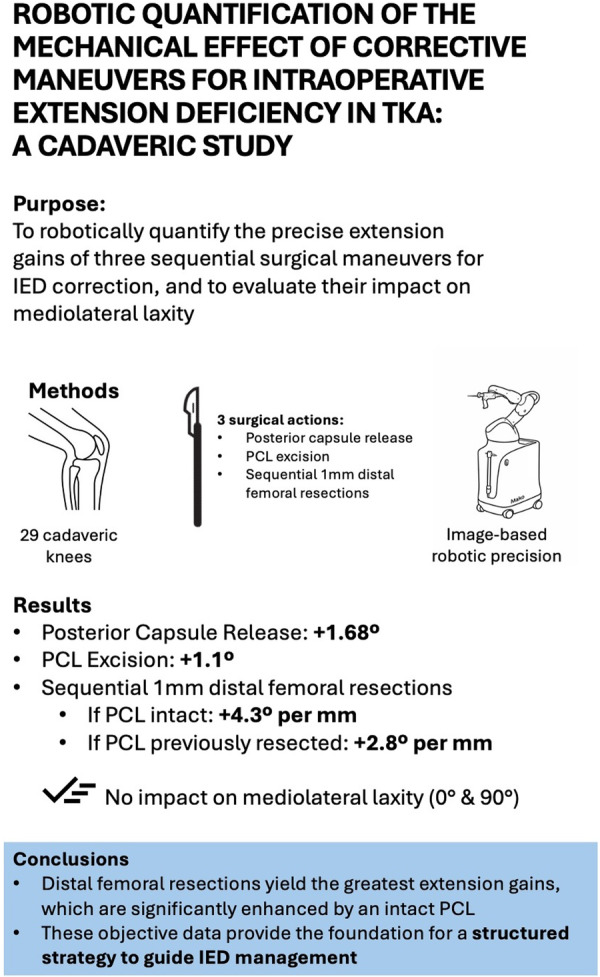

**Abbreviations**
CR: cruciate‐retaining
CT: computerised tomography
FFC: fixed flexion contracture
IED: intraoperative extension deficiency
JL: joint line
PCL: posterior cruciate ligament
PCR: posterior capsule release
SD: standard deviation
TKA: total knee arthroplasty

## INTRODUCTION

A common cause of dissatisfaction after total knee arthroplasty (TKA) is fixed flexion contracture (FFC), which is frequently observed in end‐stage knee osteoarthritis. FFC can cause quadriceps fatigue and leg length discrepancy, resulting in instability and a contracture deformity in the contralateral knee [20, 21]. However, FFC remains a significant challenge for surgeons [3, 5, 9, 19], and consequently, the postoperative prevalence of this extension deficit remains approximately 1%–17% [1].

FFC should not be viewed solely as a preoperative static condition related to chronic osteoarthritic soft‐tissue shortening. Its magnitude can change significantly after surgical exposure, osteophyte removal and bone resections [10, 27]. In particular, when aiming to achieve a tight and rectangular extension gap, the resulting gap may become different than the native state. In this context, even patients who present full extension preoperatively may develop a intraoperative extension deficit after trial component implantation.

This intraoperative extension deficit (IED) could reflect a mismatch between implant thickness and capsular compliance under standardised tensioning conditions, rather than insufficient initial bone resection or true clinical flexion contracture [9, 16]. This raises a critical question: is this IED a true clinical phenomenon, or is it an artefact of modern robotic ‘tight‐gap’ planning strategies? Accordingly, in the present study, this intraoperative residual extension deficit is conceptually distinguished from preoperative osteoarthritic FFC, and focus on quantifying the mechanical effect of different corrective manoeuvres applied after appropriate and balanced initial gap preparation. Hereafter, the concept of IED is used to describe the extension deficits observed after trial implantation, representing the actual deformity that must be addressed.

Multiple surgical actions, such as osteophyte removal, posterior capsule release, posterior cruciate ligament (PCL) excision, or increasing the distal femoral bone cut, have been proposed to prevent extension deficits [22]. However, these actions can negatively affect implant stability. PCL excision inherently alters the native kinematics, shifting the reliance for stability from the ligament to the implant's constraint profile [7]. Increasing the distal femoral resection can lengthen the surgery, thereby increasing the risk of bleeding, and, more importantly, the joint line (JL) will shift proximally, which can cause midflexion instability, anterior knee pain and a functional patella baja [4, 13].

While several studies have reported overall extension gains following these manoeuvres [12, 22, 26], the optimal sequence and the precise incremental mechanical effect of each step remain poorly quantified in the existing literature. Previous studies often lack the precision necessary to isolate the specific impact of each sequential manoeuvre. To the authors' knowledge, only one study has explored improved extension following TKA using assisted robotic technologies [6]. Robotic systems allow for highly controlled and reproducible measurements of gap changes [2, 28] which is why this technology was chosen to isolate the mechanical effect of each intervention, rather than simply assessing general implant alignment accuracy.

The main objective of this study was to quantify the incremental extension gain of different corrective surgical actions in robot‐assisted TKA, specifically, posterior capsule release, PCL excision and sequential distal femoral bone recuts for addressing intraoperative extension deficits that may arise following a tight gap‐balancing protocol. A secondary objective was to evaluate the impact of these manoeuvres on mediolateral laxity at 0° and 90° of flexion to provide objective data for managing IED in both robotic and conventional TKA.

## MATERIALS AND METHODS

### Study design

This quasi‐experimental cadaveric study was conducted in accordance with the principles of the Declaration of Helsinki. The study protocol and the use of human anatomical specimens were reviewed and approved by the Ethics Committee for Investigation of the Research and Innovation Institute in Life and Health Sciences in Central Catalonia (CEIm IRIS‐CC) (Approval Protocol Number: 24/030). Twenty‐nine knee replacements were performed on 15 fresh‐frozen lower‐limb cadaveric specimens. The remaining knee was excluded because one cadaver had previously undergone Girdlestone surgery, which made robotic knee arthroplasty impossible. Data on laterality, sex, age and degree of flexion were collected. All knees presented an intact PCL on macroscopic inspection prior to the procedure. The quality and tension of the PCL were consistent across all specimens, ensuring comparable baseline ligament conditions. Each TKA was performed using the MAKO® robotic system (Stryker), which requires a specific preoperative computerised tomography (CT) scan protocol (thin‐slice acquisition from hip to ankle) to generate a three‐dimensional (3D) model for surgical planning. A Triathlon® cruciate‐retaining (CR) total knee prosthesis (Stryker) was implanted in all knees.

### Surgical technique

One senior surgeon with extensive experience in MAKO‐assisted TKA performed all surgeries. A conventional medial parapatellar approach to the knee was employed. The PCL was present and initially preserved in all specimens. The initial degree of FFC was recorded. Following the mandatory check‐point registration and laxity assessments in both flexion and extension, the bone cuts were planned. In accordance with these plans, the tibial resection was set perpendicular to the tibial mechanical axis in the coronal plane 90°, with a standardised 3° posterior slope in the sagittal plane for all specimens. The tibial resection depth was referenced from the lateral condyle, involving a 7 mm cut from the subchondral bone for varus knees and 5 mm for valgus knees. Subsequently, the distal femoral cut was planned using a virtual gap‐balancing technique. The specific target was to achieve a rectangular extension gap with 0 mm of laxity both medially and laterally. This strict 0 mm target was chosen to ensure a precise mechanical fit, establishing a baseline where any residual soft‐tissue tightness manifests as an extension deficit. Upon completion of the bone cuts, trial components were implanted. The degree of IED, along with the degree of medial and lateral laxities at both 90° of flexion and maximum extension, was recorded for each action.

### Management of IED

**Figure 1:**
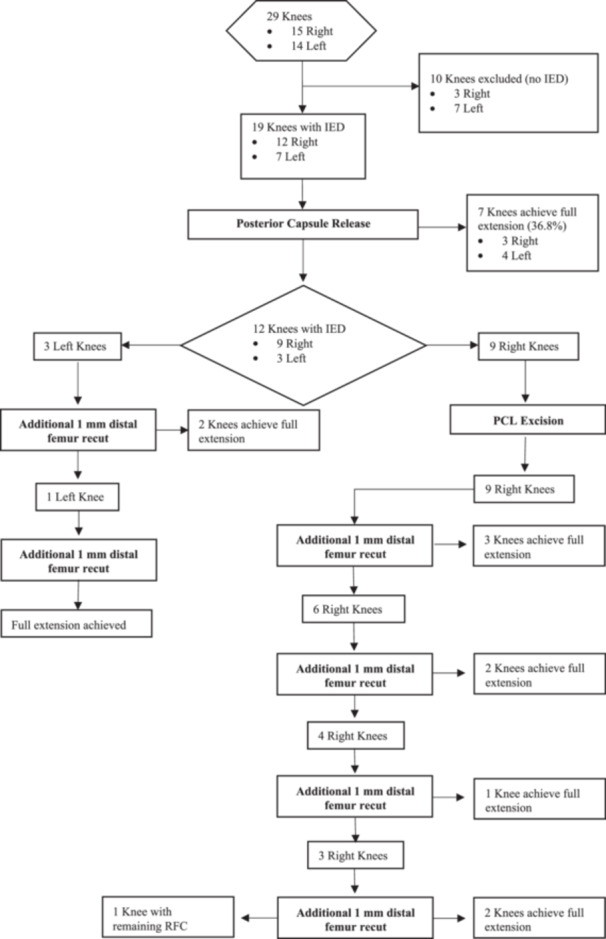
Flow diagram of specimen inclusion, group allocation and corrective actions performed.

The intraoperative extension deficit analysed in this study does not represent the true preoperative osteoarthritic FFC, but rather an intraoperative condition observed after trial component implantation under standardised gap‐balancing, which represents a clinically relevant scenario during contemporary robotic and gap‐balanced TKA. Knees with residual flexion underwent specific corrective actions to achieve full extension, defined as a value below 1° as calculated by the robotic system based on the femoral and tibial mechanical axes. To ensure reproducibility and standardise the force applied, measurements were obtained by lifting the heel (passive heel elevation), allowing the leg to extend under gravity without applying additional manual overpressure. This gravity‐assisted assessment avoids the variability of manual force, a principle emphasised by Schlegel et al. [23] for the detection of subtle flexion contractures. The initial corrective action consisted of releasing the posterior capsule (Figure 1), which was performed by osteotomizing the most posterior aspect of the femoral condyles, removing the resected bone and any associated posterior osteophytes, and thereby releasing the capsule attached to it, including the intercondylar capsule, which was carefully released with a periosteotome. To evaluate the mechanical impact of two different interventional sequences, a quasi‐experimental design based on laterality was employed. True randomisation was not performed due to logistical constraints within the cadaveric laboratory setting; instead, laterality was utilised to strictly standardize the surgical workflow and minimise procedural variability: for right knees, the second corrective action involved PCL excision, and the third and last action consisted of sequential 1 mm distal femoral bone cuts up to 4 mm, which is considered the safety margin, as supported by Minoda et al. [17]. For the left knees, the order was reversed: distal femoral additional bone recuts were performed, followed by PCL excision if complete extension was not fully achieved. After each intervention, the degree of flexion was recorded. Once full extension was achieved, no further actions were applied. Medial and lateral laxities were measured intraoperatively by the senior surgeon in both full extension and 90° flexion to assess the impact of corrective measures on TKA stability. Soft‐tissue laxity was assessed and measured intraoperatively with manual varus–valgus stress during extension and flexion and application of the force until the first mechanical stop. While distinct from tensiometer‐based quantification, this manoeuvre was performed by a single senior surgeon in order to minimise variability and enhance reliability, aiming to detect any gross changes in mediolateral stability.

### Statistical analysis

Qualitative variables are expressed as frequencies and percentages, and quantitative variables are expressed as means and standard deviations. Given the small subgroup sizes resulting from the quasi‐experimental design, the analysis of incremental mechanical gains was primarily based on descriptive quantification. After normality was assessed with the Shapiro–Wilk test, differences in continuous variables with normal distributions were evaluated according to laterality to assess mean homogeneity using Student's *t*‐test. Differences in baseline flexion values were assessed using the Kruskal–Wallis test. For variables that met the assumptions of normality, between‐group differences (such as mediolateral laxity changes) were evaluated using one‐way analysis of variance (ANOVA). A *p*‐value < 0.05 was considered statistically significant. Statistical analyses were performed using Stata version 14.0 (StataCorp LP).

## RESULTS

This cadaveric study analysed 29 knees (15 right‐sided and 14 left‐sided knees) from 10 males and 5 females, with a mean age of 77.2 ± 13.1 years (Table 1). Intraoperative extension deficit was observed in 19 of the 29 knees following provisional prosthesis implantation, with a mean deficit of 6.6° ± 4.9° and a range of 2°–21° (Table 2). The remaining 10 knees were excluded because they had no IED. Stratifying by severity, 9 knees presented an IED of <5°, 6 knees presented an IED between 5° and 10°, and 4 knees had an IED of >10°.

**Table 1:** Demographic characteristics of the cadaveric specimens included in the study. (*n* = 29).

Variable	*n* (%)
Sex	
Male	10 (66.7%)
Female	5 (33.3%)
Age (mean ± SD)	77.2 ± 13.1
Laterality	
Right	15 (51.7%)
Left	14 (48.3%)

**Table 2:** Distribution of intraoperative sagittal deformities (intraoperative extension deficit, neutral and recurvatum) in the study sample; only knees with IED (*n* = 19) were included for analysis.

Distribution of intraoperative sagittal deformities after trial implantation	*n*	Degrees (mean ± SD)
Negative flexion (recurvatum)	2	−4.0 ± 1.4
Neutral	8	0.3 ± 0.7
Intraoperative extension deficit	19	6.6 ± 4.8
Total	29	

### First corrective action: Posterior capsule release

The initial corrective action in both knees involved release of the posterior capsule (Table 3). This intervention achieved full extension in 7 of the 19 knees (36.8%). On average, posterior capsule release improved extension by 1.68° ± 1.11°. Notably, the standard deviation is nearly equal to the mean, reflecting a high inter‐specimen variability in the mechanical response to this soft‐tissue release. When stratified by laterality, the right knees showed a slight improvement in extension compared with the left knees (1.58° right vs. 1.86° left), with no statistically significant difference (*p* = 0.618). After this action, 12 knees (9 right and 3 left) required further corrective measures. The discrepancy in sample size between groups regarding laterality was incidental and only related to the availability of specimens with IED after trial implantation.

**Table 3:** Extension gain (degrees) and achievement of full extension after sequential corrective manoeuvres in knees with intraoperative extension deficit (IED).

Action	*n*	Degrees (mean ± SD)	Number of knees achieving full extension
Baseline sagittal alignment			
Right	15	0.53 ± 5.37	
Left	14	−2.36 ± 4.18	
Total	*29*	−0.86 ± 4.97	
Posterior capsule release			
Right	12	−1.58 ± 1.08	3
Left	7	−1.86 ± 1.21	4
Total	*19*	−1.68 ± 1.11	7
Right: PCL excision	9	−1.11 ± 1.27	0
Left: 1st distal femoral cut (1 mm)	3	−4.33 ± 0.58	2
Total	*12*	−1.92 ± 1.83	2
Right: 1st distal femoral cut (1 mm)	9	−2.78 ± 0.83	3
Left: 2nd distal femoral cut (2 mm)	1	−2.00 ± 0.00	1
Total	*10*	−2.70 ± 0.82	4
Right: 2nd distal femoral cut (2 mm)	6	−4.00 ± 3.35	2
Left: No action	—	—	—
Right: 3rd distal femoral cut (3 mm)	4	−3.50 ± 3.11	1
Left: No action	—	—	—
Right: 4th distal femoral cut (4 mm)	3	−2.67 ± 3.79	2
Left: No action	—	—	—

### Second corrective action

For the right knees, the second corrective action involved PCL excision, resulting in an average extension improvement of 1.1° ± 1.3°. However, none of the nine right knees achieved full extension with this intervention.

In contrast, the second action for the left knees, consisting of sequential 1 mm distal femoral resections performed with an intact PCL, improved extension by 4.3° ± 0.6°, achieving full extension in two of the three left knees. The remaining left knee achieved full extension with an additional 1 mm bone cut.

### Third corrective action

For the nine remaining right knees, the third action consisted of sequential 1 mm distal femoral resections up to a maximum of 4 mm, performed after PCL excision. Notably, the first 1 mm distal femoral bone resection improved extension by 2.8 ± 0.8°. Full extension was achieved in three knees with a 1 mm resection, two with a 2 mm resection, one with a 3 mm resection, and finally, two with a 4 mm resection. One knee retained an IED despite a 4 mm resection. With respect to the left knees, the third action consisted of excision of the PCL, although it was not required since all the left knees reached full extension after the second action.

### Laxity measurements

Medial and lateral laxities were assessed intraoperatively in full extension and at 90° flexion. Overall, none of the corrective actions resulted in a significant increase in mediolateral laxity. Posterior capsule release, PCL excision, and sequential distal femoral resections maintained stable medial and lateral balance throughout the procedure. As shown in Table 4, the mean changes in laxity were minimal and close to zero in both extension and flexion. Statistical analysis revealed no significant differences in medial or lateral stability between groups (all *p* > 0.1), confirming that none of the corrective actions compromised TKA stability.

**Table 4:** Values represent the mean change in laxity from baseline; positive values indicate increased laxity, and negative values indicate decreased laxity.

Action	Δ Medial Ext (mm)	Δ Lateral Ext (mm)	Δ Medial Flex (mm)	Δ Lateral Flex (mm)
Posterior capsule release (PCR)	0.00 ± 0.52	−0.14 ± 0.24	−0.36 ± 0.38	−0.29 ± 0.39
PCR + distal femoral bone cut	−0.25 ± 0.52	0.16 ± 0.41	−0.42 ± 0.49	−0.25 ± 0.94
PCR + PCL excision + distal femoral bone cut	−0.50 ± 0.87	−0.50 ± 0.87	−0.50 ± 0.50	−0.83 ± 0.76
*p*	0.414	0.148	0.895	0.477

*Note*: *p*‐values refer to one‐way analysis of variance comparing the three corrective conditions for each laxity parameter (medial extension, lateral extension, medial flexion and lateral flexion).

## DISCUSSION

The main finding of the present study was that sequential posterior capsule release, PCL excision and distal femoral recuts performed with robotic assistance each provided measurable and progressive improvements in knee extension, allowing full correction of IED in nearly all specimens. One specimen could not be fully corrected due to a more pronounced baseline IED of 21° (the maximum in the observed range). Rather than establishing a definitive clinical guideline, these objective findings provide the foundation for a conceptual decision‐making framework for IED correction that may serve as a guide in both robotic and conventional TKA.

### Posterior capsule release

In the present study, posterior capsule release yielded an average extension gain of 1.68° ± 1.1°, which was sufficient to correct IED in more than one‐third of the knees (36.8%). These results demonstrate that posterior capsule release is a viable initial step for addressing mild IED, particularly when combined with the resection of posterior condylar osteophytes, which is often performed simultaneously. Interpretation of these findings within the context of the current study population is essential. While posterior capsule release alone achieved meaningful correction, it cannot be conclusively asserted that it should always be the preferred starting manoeuvre. Instead, its efficacy in mild contractures suggests that it may be an effective initial action in the course toward complete IED correction.

These findings partially align with those of prior studies. For example, Okamoto et al. [18] emphasised the importance of releasing the posterior capsule in improving the extension gap. Marfo et al. [14] further suggested that soft‐tissue release alone might suffice in many cases, cautioning against the tendency to immediately increase distal femoral resection depth upon encountering FFC. Nevertheless, caution is advised about suggesting that posterior capsule release is universally superior, as the present study primarily sought to quantify its extension gain rather than evaluate its role relative to other interventions.

### Distal femoral resection

A substantial extension gain of 4.3° per millimetre with distal femoral resection was observed after posterior capsule release. This result significantly exceeds the gains reported in earlier studies. For example, an extension gain of 3.6° for every 2 mm of resection was reported by Smith et al. [26]. It is hypothesised that this amplified effect in the present series may be related to the strict 0‐mm rectangular extension gap targeting philosophy used during robotic planning. Imposing a 0‐mm gap creates a state of high capsular tension. Under this condition, it is theorised that the intact PCL may act as a rigid posterior tether; consequently, any minimal bony resection releases significant mechanical tension, resulting in a magnified angular gain. Interestingly, when distal femoral resections were performed after PCL excision, a decrease in the observed extension gain to 2.8° per millimetre was noted, making these findings more consistent to those of previous studies, such as those by Matziolis et al. [15], who reported gains of 2.2° ± 0.3° per millimetre with posterior‐stabilised implants. This suggests that the mechanical influence of the PCL, when intact, may increase the effectiveness of distal femoral resection. In contrast, its excision may reduce the mechanical tension necessary to achieve maximal extension gain.

Moreover, an extension gain of 2° ± 0.6° per millimetre using the ROSA robotic system was reported in the most recent study on this topic by Hishimura et al. [6], whose work was conducted with posterior‐stabilised implants (Persona PS) and therefore with systematic PCL resection. The greater extension gains observed in the current study could be attributed to a combination of factors, including the use of a cadaveric model rather than a clinical setting, the specific implant systems employed, or potentially the differences between direct and indirect robotic cutting mechanisms.

### PCL excision

The extension gain achieved solely through PCL excision in the present study was limited to an average of 1.1°, which contrasts with the greater gains reported by other studies. An average extension gain of 2.5° ± 2.2° was observed following PCL excision by Kim et al. [8], whereas an increase of 2.9° ± 1.6° was reported by Kayani et al. [7]. Although PCL excision can help correct IED, it should be performed with caution, as it alters biomechanics of the knee and imposes constraints more significantly than other manoeuvres. In the current series, greater extension gains were obtained after distal femoral recuts while preserving the PCL, suggesting that this step may be more effective and biomechanically conservative. However, in cases with low patellar height or mild IED in which additional distal resection may not be advisable [24], the combination of posterior capsular release and PCL excision can represent a reasonable alternative. This choice must be made with the understanding that the level of prosthetic constraint will consequently be altered.

### Effect of corrective actions on laxity

With respect to mediolateral stability, none of the corrective manoeuvres produced gross changes in medial or lateral laxity, either in full extension or in 90 degrees of flexion. These findings are consistent with previous reports showing that posterior capsular release and additional distal femoral resections have only minimal effects on coronal plane stability [7, 15]. These findings support the conclusion that the corrective steps evaluated can be safely performed without compromising TKA stability in both full extension and in 90 degrees of flexion.

### Structured strategy for IED correction

**Figure 2:**
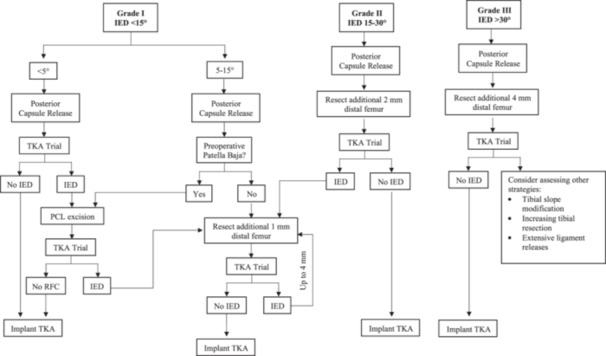
Proposed structured strategy for the management of intraoperative extension deficiency (IED) in total knee arthroplasty, stratified according to the Lombardi classification.

Based on the incremental gains quantified in this study, a structured strategy is proposed for IED correction, stratified according to the Lombardi classification and consistent with previously published approaches (Figure 2) [8, 14, 22]. Although the Lombardi classification was originally described for preoperative FFC, it is used here as a pragmatic tool to stratify the severity of IED. The proposed strategy incorporates the precision of the robotic measurements to differentiate between two pathways within the mild IED range (<15°), allowing for more tailored corrections. Crucially, this specific range represents the most frequent scenario encountered in primary TKA [21], underscoring the daily clinical relevance of this approach.

However, this framework should not be interpreted as a validated clinical guideline, given the limitations of a cadaveric sample size. Instead, it serves as a conceptual decision‐making guide intended to assist intraoperative judgement by estimating the relative mechanical impact of each corrective action. Although derived from a cadaveric model, this strategy is intended to complement rather than replace existing recommendations, and its principles may also be applied in conventional TKA. Further clinical studies are needed to validate its applicability, particularly in cases involving severe deformities, which were underrepresented in the evaluated sample.

### Advantages of MAKO robotic precision

A major strength of this study lies in the use of robotic technology, which provides a data‐driven approach that minimises inter‐observer variability. The MAKO system achieved high accuracy in both resection depth and extension‐angle measurement, enabling precise quantification of the effects of each corrective manoeuvre. This level of reproducibility is challenging to achieve with manual techniques and increases the reliability of the proposed strategy. Previous work similarly demonstrated that MAKO achieves bone resection within 1 mm of the surgical plan and maintains alignment deviations below 1° [11, 25]. Such precision is particularly relevant in flexion contracture correction, where slight variations in resection depth may significantly influence extension gains [6].

Furthermore, while robotic precision allows for exact measurement, it also prompts a reevaluation of modern surgical philosophies. The high incidence of IED in the evaluated sample raises the possibility that the rigid pursuit of a 0‐mm rectangular gap may inadvertently ‘create’ an extension deficit by over‐tensioning the native soft‐tissue envelope.

### Limitations

This study has certain limitations. First, it was conducted on cadaveric specimens and does not fully replicate the clinical setting, particularly due to the absence of active muscle tone and dynamic stabilisers. Nevertheless, the use of 29 whole fresh‐frozen cadaveric specimens represents a substantial cohort for this highly specific biomechanical model, providing a highly realistic anatomical environment to evaluate incremental gap changes. Furthermore, the stepwise sequence of corrective manoeuvres could not be realistically reproduced in vivo, because performing each release or resection before implant placement would considerably increase surgical time and expose patients to unnecessary risks. Cadaveric testing, therefore, provided the only feasible environment for analysing the incremental contribution of each corrective action.

Second, the study utilised a quasi‐experimental, non‐randomised sequence allocation based on laterality. Unexpectedly, an uneven incidence of IED between left and right specimens was observed. This resulted in small subgroup sizes for certain comparisons (e.g., the group undergoing distal femoral resections first). Consequently, robust comparative statistical analyses were limited, and the focus was appropriately placed on descriptive quantification of the mechanical gains.

Third, coronal laxity was assessed via manual stress without a tensioning device. Furthermore, measurements were restricted to static positions at 0° and 90°, resulting in a lack of dynamic or mid‐flexion stability assessment. However, evaluating dynamic mid‐flexion instability was not an objective of this study.

Fourth, the surgeries were performed with the exclusive use of cruciate‐retaining (CR) implants. The magnitude of the extension gains observed, particularly those following distal femoral resections, would likely differ if posterior‐stabilised (PS) implants had been used, given the routine sacrifice of the PCL inherent to that design

Finally, the protocol focused specifically on capsular release, PCL excision and distal femoral recuts. Consequently, there was a lack of assessment regarding tibial slope modification, which is another known surgical variable that can influence flexion‐extension gaps. However, this omission was a deliberate methodological choice to maintain sample homogeneity and avoid introducing confounding variables into this specific biomechanical analysis.

## CONCLUSIONS

This study highlights the value of robotic quantification for assessing the mechanical impact of sequential corrective actions for IED in robot‐assisted TKA. Posterior capsule release emerged as an impactful initial step, particularly for mild contractures, whereas distal femoral resections provided greater extension gains when the PCL was intact, improving the effectiveness of this approach.

Despite the limitations of a cadaveric study, the proposed strategy for IED correction offers a systematic approach that could also be applied to conventional TKA. Future research should validate these findings in clinical settings and further explore the role of PCL integrity in IED correction efficacy and evaluate the influence of other variables to establish definitive clinical guidelines.

## AUTHOR CONTRIBUTIONS

All authors contributed to the experimental phase of the study. Joan Leal‐Blanquet and Juan Ignacio Erquicia were responsible for the study conception and design. Material preparation and data collection were performed by Eric Camprubí, Berta Gasol, Santiago Bonduel, Albert Pons‐Riverola and Ángela Zumel. Statistical analysis was performed by Ángela Zumel. The first draft of the manuscript was written by Albert Pons‐Riverola, and it was critically reviewed and revised by Joan Leal‐Blanquet and Juan Ignacio Erquicia. All authors read and approved the final manuscript.

## CONFLICT OF INTEREST STATEMENT

Joan Leal‐Blanquet is a consultant for Stryker. The remaining authors declare no conflicts of interest.

## ETHICS STATEMENT

Comitè d'Ètica d'Investigació amb Medicaments de l'Institut de Recerca i Innovació en Ciències de la Vida i la Salut a la Catalunya Central (CEIm IRIS‐CC). Approval Number 24/030.

## Data Availability

The data that support the findings of this study are available from the corresponding author upon reasonable request.
