# Correction to “Robotic quantification of the mechanical effect of corrective manoeuvres for intraoperative extension deficiency in total knee arthroplasty: A cadaveric study”

**DOI:** 10.1002/jeo2.70970

**Published:** 2026-11-03

Pons‐Riverola A, Erquicia JI, Camprubí E, Gasol B, Zumel Á, Bonduel S, et al. Robotic quantification of the mechanical effect of corrective manoeuvres for intraoperative extension deficiency in total knee arthroplasty: a cadaveric study. *J Exp Orthop*. 2026;13:e70740. 10.1002/jeo2.70740

In the “ACKNOWLEDGEMENTS” section, the text “The authors have no funding to report.” was incomplete. This should have read: “The authors have no funding to report. This work forms part of the doctoral thesis of Albert Pons‐Riverola at the Department of Surgery, Universitat Autònoma de Barcelona, Barcelona, Spain.”

We apologize for this error.

